# The muscle dysmorphia–eating disorders link as a public health concern: an epidemiological study of symptom dimensions and social isolation

**DOI:** 10.3389/fpubh.2026.1805859

**Published:** 2026-04-14

**Authors:** Francesco Leonforte, Michelangelo Mercogliano, Vito Nicosia, Manuela Lo Bianco, Roberta Leonardi, Andrea Cavallaro, Martino Ruggieri, Antonio Mistretta

**Affiliations:** 1 Department of Integrated Hygiene, Organizational, and Service Activities (Structural Department), Health Management, University Hospital Polyclinic "G. Rodolico - San Marco", Catania, Italy; 2 National PhD Programme in One Health Approaches to Infectious Diseases and Life Science Research, Department of Public Health, Experimental and Forensic Medicine, University of Pavia, Pavia, Italy; 3 Department of Medical, Surgical Sciences and Advanced Technologies "G.F. Ingrassia", University of Catania, Catania, Italy; 4 Unit of Pediatric Clinic, Department of Clinical and Experimental Medicine, University of Catania, Catania, Italy; 5 Neonatal Intensive Care Unit, AOU Policlinico G. Rodolico San Marco, Catania, Italy; 6 Postgraduate Training Programme in Pediatrics, Department of Clinical and Experimental Medicine, University of Catania, Catania, Italy; 7 Department of General Surgery and Medical-Surgical Specialties, University of Catania, Catania, Italy; 8 Scientific Communication Service, National Institute of Public Health (Istituto Superiore di Sanità), Roma, Italy

**Keywords:** EAT-26, eating disorders, lifestyle, MDDI, muscle dysmorphia, sociodemographic factors, university students

## Abstract

**Background:**

Eating disorders (EDs) represent a growing public health concern among university students, who face critical developmental transitions and increasing pressure from “fitness culture.” This study examined the complex relationship between muscle dysmorphia (MD) symptomatology and ED risk, analyzing how specific psychopathological dimensions interact with socio-demographic variables and lifestyles.

**Methods:**

A cross-sectional study was conducted on a sample of 2,001 students from University of Catania using a web-based survey. The primary outcome was the risk of disordered eating attitudes, assessed via the Eating Attitudes Test-26 (EAT-26). The main explanatory variable was MD symptomatology, measured using the Muscle Dysmorphia Disorder Inventory (MDDI), analyzed both as a cumulative clinical risk (score >39) and through its specific subscales: Drive for size (DFS), Appearance intolerance (AI), and Functional impairment (FI). Multivariable logistic regression models were fitted to identify independent predictors.

**Results:**

The presence of clinical MD symptoms was identified as one of the strongest independent predictors of ED risk (OR = 2.94; 95% CI: 2.03–4.25). A sensitivity analysis on the MDDI subscales revealed divergent pathways: AI (OR = 1.18; 95% CI: 1.14–1.23) and FI (OR = 1.22; 95% CI: 1.17–1.28) were positive drivers of eating risk, whereas DFS showed a significant inverse association (OR = 0.93; 95% CI: 0.89–0.96). Sociodemographic analysis indicated that students aged 18–23, those living alone, and those who preferred not to disclose their gender (OR = 2.07; 95% CI: 1.15–3.73) were at significantly higher risk. Conversely, living with a partner (OR = 0.24; 95% CI: 0.13–0.42) or parents (OR = 0.39; 95% CI: 0.28–0.54) offered robust protection. Regarding lifestyle, intense sports practice was associated with a higher risk of EDs (OR = 1.50; 95% CI: 1.19–1.90).

**Conclusion:**

The findings confirm that MD symptomatology acts as a specific “gateway” to disordered eating but highlights a crucial distinction: the risk is driven by body image intolerance and social impairment rather than the ambition for muscle growth itself, which may be protective. These results suggest that university prevention strategies should focus on vulnerable phenotypes—specifically younger students, those socially isolated, and gender minorities—and screen for the distress accompanying fitness goals rather than the athletic discipline itself.

## 1 Introduction

The transition to university is identified as a high-risk period, as significant changes in lifestyle, increased academic pressure, and greater independence during this developmental stage can exacerbate the risk for psychopathological behaviors (1, 2). Recent epidemiological data indicate that the prevalence of eating disorders (EDs) risk in university populations has risen sharply, with estimates ranging from 15% to over 30% depending on the cultural context and assessment tools used (3, 4). Similar epidemiological trends emphasizing the importance of screening and prevention have been observed in other clinical populations (5). Particularly, emerging evidence suggests a gender-neutral shift in body image disturbance, driven by the internalization of an athletic and mesomorphic ideal (6, 7). In this context, although clinical research has historically focused attention on the female gender for the management of specific health and esthetic wellness needs, the emerging literature on body image disorders highlights the necessity of moving beyond traditional paradigms to understand how ‘fitness culture’ transversally influences all gender identities (8). In this evolving landscape is placed muscle dysmorphia (MD) (9), a subtype of body dysmorphic disorder (BDD) characterized by a pathological preoccupation with perceived physical smallness, despite significant muscular development (9). Clinically, it manifests through compulsive resistance training, rigid dieting, and psychosocial impairment, often leading to the abuse of performance-enhancing drugs (10, 11). Psychologically, MD severity interacts with distinct personality traits: while competitive bodybuilders exhibit higher levels of narcissism, non-competing individuals show more pronounced depressive symptomatology (12). Ultimately, MD is frequently comorbid with anxiety and other EDs, reflecting deep-seated vulnerabilities in self-esteem and the internalization of unrealistic muscular ideals (12). Beyond psychopathological drivers, in existing literature MD reveals a complex interplay between several sociodemographic variables, offering a critical framework for identifying vulnerable cohorts. A consistent gender-based risk appears to be rooted in the masculine ideal. Although men consistently exhibit higher clinical risk and symptom scores, research indicates that the prevalence gap is narrowing among adolescents (13, 14), suggesting a broadening of sociocultural pressures (13, 14). Notably, age serves as a significant correlate, with adolescents and young adults showing a higher susceptibility to MD symptoms. This trend is driven by the intensive identity formation and heightened sensitivity to peer and social media influence characteristic of youth (15). Conversely, socioeconomic status appears to have a negligible impact on MD development. Education, however, may serve as a slight protective factor, as higher educational attainment is occasionally linked to lower odds of MD. This may be due to increased health literacy or a more diversified range of identity-building markers beyond physical appearance (16).

The Muscle Dysmorphic Disorder Inventory (MDDI) serves as a gold-standard instrument, operationalizing the condition into three distinct dimensions: Drive for size (DFS), Appearance intolerance (AI), and Functional impairment (FI). Specifically, DFS evaluates the desire to increase musculature and the perception of being less muscular than desired, reflecting the drive to increase the size of specific body parts. AI captures body dissatisfaction and intolerance toward one’s appearance, encompassing negative beliefs about the body, anxiety regarding physical exposure, and the avoidance of situations where the body might be judged. Finally, FI measures the degree to which these symptoms interfere with daily functioning (17, 18). This includes excessively rigid training routines, the sacrifice of social or occupational activities, and marked distress when unable to adhere to exercise regimens. Recent validation studies, including those in Italian populations, suggest that these subscales may have differential associations with general psychopathology (14, 17). While DFS might reflect a goal-oriented behavior common in sports, AI and FI are more strongly correlated with distress and compulsive symptoms. Understanding these nuances is essential for developing targeted prevention strategies on university campuses.

Theoretical frameworks often cite the ubiquity of “fitness culture” and image-centric social media platforms as relevant environmental factors in the proliferation of MD symptoms among general university cohorts (19, 20). While the literature suggests that exposure to digital environments (e.g., Instagram, TikTok) may correlate with body dissatisfaction and “fitspiration” internalization in the broader population (21–23).

However, the specific mechanisms by which these muscularity-oriented concerns translate into generalized ED risk remain under-explored. It is debated whether the “drive” for muscularity itself is maladaptive, or if the risk stems primarily from the FI and intolerance of one’s body image (24, 25). Notably, as this study does not directly measure social media exposure, the focus remains on the internal psychopathological dimensions of MD and their association with ED risk (14, 17).

The primary objective of this study was to clarify the relationship between MD symptomatology and disordered eating attitudes in a university student population, while also assessing the contribution of socio-demographic and lifestyle factors to EDs risk, assessed using the Eating Attitudes Test-26 (EAT-26). A secondary objective was to examine the contribution of muscle dysmorphia symptomatology, measured using the MDDI, both as a cumulative risk score and through its individual subscales, in predicting disordered eating attitudes in this demographic. In line with existing validation literature, we hypothesized that AI and FI would be the primary positive drivers of EDs risk, given their stronger correlation with psychological distress and compulsive symptoms. Whether DFS constitutes a risk factor or, conversely, reflects an adaptive form of athletic motivation remains an open empirical question; therefore, its specific contribution was explored.

## 2 Methods

### 2.1 Study design and participants

This cross-sectional study was conducted between September and October 2025 among university students at the University of Catania. The study protocol was approved by the local Ethics Committee of Catania 1 (Protocol no. no.55462 n.56/2025-PAR). Participation in the study was entirely on a voluntary and anonymous basis. Participants were recruited via a web-based survey and provided informed consent prior to participation. The online questionnaire was developed using Google Form (© 2025 Google, Mountain View, CA, USA) and widespread via a QR code posted in university classrooms reserved exclusively for medical students. The full survey instrument is available in the Supplementary Materials.

### 2.2 Variables

The primary outcome was the risk of disordered eating attitudes, assessed using the EAT-26 (26). Participants were classified as “at risk” (total score >19) or “not at risk” (total score ≤19) based on established cut-offs. The main explanatory variable was MD symptomatology, measured using the Italian validation of the MDDI (17, 18). The high levels of MD symptoms were assessed using the validated cut-off of >39 on the MDDI total score. A score > 39 has been proposed as a clinical threshold and employed in previous studies (14, 27). Secondary analyses considered MDDI as a continuous variable, examining both the total score and its three subscales: DFS, AI, and FI. Covariates included sociodemographic and lifestyle factors: age group, gender, educational level, degree course, smoking status, residence status, living arrangement, and family size. Physical activity was categorized based on frequency: participants training ≥4 times per week for a minimum of 6 months per year were classified as “sporty,” while those training ≤3 times per week or for <6 months per year, were classified as “non-sporty” (28).

### 2.3 Statistical analysis

Descriptive statistics were used to summarize participant characteristics and the distribution of EAT-26 risk. Categorical variables were compared using Chi-squared tests. The normality of continuous MDDI scores was assessed using the Shapiro–Wilk test and graphical inspection; given the non-normal distribution, differences were evaluated using the Wilcoxon rank-sum test, with results reported as medians and interquartile ranges.

The association between EAT-26 risk and variables was first estimated using univariable logistic regression models. Subsequently, a main multivariable logistic regression model was fitted to assess the independent association between high ED symptoms (MDDI >39) and disordered eating risk. To further dissect this relationship, additional multivariable models were constructed considering, instead of the binary MDDI variable:

1. The continuous MDDI total score;
2. The DFS MDDI subscale;
3. The AI MDDI subscale;
4. The FI MDDI subscale.

A sensitivity analysis was performed by including all three MDDI subscales in the same model to confirm results. All multivariable models were adjusted for age, gender, education, degree course, smoking, residence, living situation, family size, and sport practice. Results are reported as Odds Ratios (ORs) with 95% Confidence Intervals (95% CIs). All analyses were performed using Stata/MP version 18 (StataCorp LLC, College Station, TX, USA), considering a two-sided *p*-value < 0.05 as statistically significant.

## 3 Results

The analysis of sociodemographic characteristics revealed distinct patterns of association (Table 1). Regarding age, the 24–29 age group showed a negative association with the outcome (OR = 0.71; 95% CI: 0.57–0.88) compared to the youngest cohort (18–23 years). While no significant association was observed for male students compared to females, the subgroup of participants who chose not to disclose their gender (*n* = 58) exhibited a positive association (OR = 3.14; 95% CI: 1.84–5.37).

**Table 1:** Sociodemographic characteristics and lifestyle habits of the study population, stratified by risk of disordered eating attitudes.

Variable		EAT-26			χ^2^ *p*-value	Odds ratio	*p*-value	95% CI
		Not at risk	At risk	Total				
N°		1,485	516	2,001				
Age group	18–23	757 (51.0%)	302 (58.5%)	1,059 (52.9%)	<0.001	Ref		
	24–29	591 (39.8%)	168 (32.6%)	759 (37.9%)		0.71	0.002	0.57–0.88
	30+	137 (9.2%)	46 (8.9%)	183 (9.2%)		0.84	0.347	0.59–1.21
Gender	Female	758 (51.0%)	258 (50.0%)	1,016 (50.8%)	<0.001	Ref		
	Male	699 (47.1%)	228 (44.2%)	927 (46.3%)		0.95	0.685	0.78–1.78
	Prefer not to say	28 (1.9%)	30 (5.8%)	58 (2.9%)		3.14	<0.001	1.84–5.37
Highest educational level	High school	971 (65.4%)	283 (54.9%)	1,254 (62.7%)	<0.001	Ref		
	Bachelor’s degree	407 (27.4%)	187 (36.2%)	594 (29.7%)		1.58	<0.001	1.27–1.96
	Master’s degree or higher	107 (7.2%)	46 (8.9%)	153 (7.6%)		1.47	0.040	1.02–2.14
Current degree course level	Bachelor’s degree	801 (53.9%)	270 (52.3%)	1,071 (53.5%)	0.818	Ref		
	Master’s degree	579 (39.0%)	208 (40.3%)	787 (39.4%)		1.07	0.552	0.86–1.31
	Postgraduate (Special)	105 (7.1%)	38 (7.4%)	143 (7.1%)		1.07	0.725	0.72–1.59
Smoker status	Non-smoker	755 (50.8%)	219 (42.44%)	974 (48.7%)	0.001	Ref		
	Smoker	730 (49.2%)	297 (57.56%)	1,027 (51.3%)		1.40	0.001	1.15–1.72
Residence status	Erasmus	12 (0.8%)	11 (2.13%)	23 (1.1%)	0.001	Ref		
	Commuter	132 (8.9%)	55 (10.66%)	187 (9.4%)		0.45	0.078	0.19–1.09
	Living away from home	627 (42.2%)	173 (33.53%)	800 (40.0%)		0.30	0.005	0.13–0.69
	Resident	714 (48.1%)	277 (53.68%)	991 (49.5%)		0.42	0.042	0.18–0.97
Living situation	Alone	364 (24.5%)	210 (40.7%)	574 (28.7%)	<0.001	Ref		
	With partner	125 (8.4%)	17 (3.29%)	142 (7.1%)		0.24	<0.001	0.14–0.40
	With parents	430 (29.0%)	115 (22.29%)	545 (27.2%)		0.46	<0.001	0.35–0.60
	With friends/flatmate	566 (38.1%)	174 (33.72%)	740 (37.0%)		0.53	<0.001	0.42–0.68
Number of family members	1–3	765 (51.5%)	313 (60.7%)	1,078 (53.9%)	<0.001	Ref		
	4 or more	720 (48.5%)	203 (39.3%)	923 (46.1%)		0.69	<0.001	0.56–0.84
Physical activity level	No sporty	821 (55.3%)	209 (40.5%)	1,030 (51.5%)	<0.001	Ref		
	Sporty	664 (44.7%)	307 (59.5%)	971 (48.5%)		1.82	<0.001	1.48–2.23

Data are presented as number and column percentage within each group. EAT-26: Eating Attitudes Test-26. Participants were classified as “At risk” if the EAT-26 total score was >19. OR: Odds ratio; 95% CI: 95% Confidence interval; Ref: Reference category used for the logistic regression analysis. *χ*^2^^ *p*-value: Significance level derived from the Chi-squared test for comparison between “Not at risk” and “At risk” groups.

Considering educational level, students holding a Bachelor’s degree (OR = 1.58; 95% CI: 1.27–1.96) or a Master’s degree (OR = 1.47; 95% CI: 1.02–2.14) showed higher odds of disordered eating attitudes compared to those with a high school diploma.

In terms of housing and family context, students living alone (reference group) showed the highest prevalence of disordered eating. Conversely, cohabiting with a partner yielded a strong inverse association (OR = 0.24; 95% CI: 0.14–0.40), as did living with parents (OR = 0.46; 95% CI: 0.35–0.60) or with flatmates (OR = 0.53; 95% CI: 0.42–0.68). Similarly, a larger family size (4 or more members) was negatively associated with the outcome (OR = 0.69; 95% CI: 0.56–0.84). Furthermore, compared to Erasmus students, those classified as ‘living away from home’ (OR = 0.30; 95% CI: 0.13–0.69) and permanent residents (OR = 0.42; 95% CI: 0.18–0.97) demonstrated significantly lower odds.

Lifestyle factors emerged as significant correlates. Smokers exhibited a positive association (OR = 1.40; 95% CI: 1.15–1.72) compared to non-smokers. Physical activity levels also indicated a significant trend: “Sporty” students (training 4 or more times/week) showed a positive association with scoring above the EAT-26 cut-off compared to “Non-sporty” (OR = 1.82; 95% CI: 1.48–2.23).

Tables 2, 3 details the association between MD symptomatology and EDs. The presence of clinical ED symptoms (MDDI >39) was strongly correlated with EAT-26 risk (OR = 3.36; 95% CI = 2.42–4.67). Students classified as “at risk” for EDs (EAT-26 > 19) reported significantly higher median scores across the entire MDDI spectrum (total score, DFS, AI, and FI) (*p* < 0.001 for all comparisons).

**Table 2:** Comparison of MDDI clinical cut-off prevalence between participants at risk and not at risk of EDs.

Variable		EAT-26			*χ*^2^ *p*-value	Odds ratio	*p*-value	95% CI
		Not at risk	At risk	Total				
N°		1,485	516	2,001				
MDDI clinical risk	No	1,407 (94.7%)	435 (84.3%)	1,842 (92.1%)	<0.001	Ref		
	Yes	78 (5.3%)	81 (15.7%)	159 (7.9%)		3.36	<0.001	2.42–4.67

Data are presented as number and column percentage within each group. EAT-26: Eating Attitudes Test-26. Participants were classified as “At risk” if the EAT-26 total score was >19. MDDI Clinical Risk: Identification of ED symptomatology based on a total score >39. MDDI: Muscle Dysmorphia Disorder Inventory. *χ*2^ *p*-value: Significance level derived from the Chi-squared test for comparison between “Not at risk” and “At risk” groups. Ref: Reference category used for the logistic regression analysis.

**Table 3:** Differences in MDDI scores between groups and univariable association with disordered eating risk.

Variable	EAT-26			Test Mann–Whitney	Odds ratio	*p*-value	95% CI
	Not at risk	At risk	Total				
N°	1,485	516	2001				
MDDI total score	22 (IQR: 10)	30 (IQR: 11)	25 (IQR: 12)	*p* ≤ 0.001	1.10	<0.001	1.08–1.11
MDDI—DFS	8 (IQR: 6)	11 (IQR: 6)	9 (IQR: 6)	*p* ≤ 0.001	1.11	<0.001	1.08–1.14
MDDI—AI	8 (IQR: 4)	10 (IQR: 5)	8 (IQR: 5)	*p* ≤ 0.001	1.21	<0.001	1.17–1.24
MDDI—FI	5 (IQR: 4)	9 (IQR: 3)	7 (IQR: 5)	*p* ≤ 0.001	1.29	<0.001	1.25–1.34

Data are presented as Median (Interquartile Range, IQR). *p*-value (Mann–Whitney): Significance level derived from the Mann–Whitney U test comparing “Not at risk” vs. “At risk” groups. EAT-26: Eating Attitudes Test-26. Participants were classified as “At risk” if the EAT-26 total score was > 19. MDDI: Muscle Dysmorphia Disorder Inventory; DFS: Drive for size; AI: Appearance intolerance; FI: Functional impairment. OR: Odds ratio calculated using univariable logistic regression models, indicating the increase in risk for each unit increase in the MDDI score. 95% CI: 95% Confidence interval.

**Figure 1:**
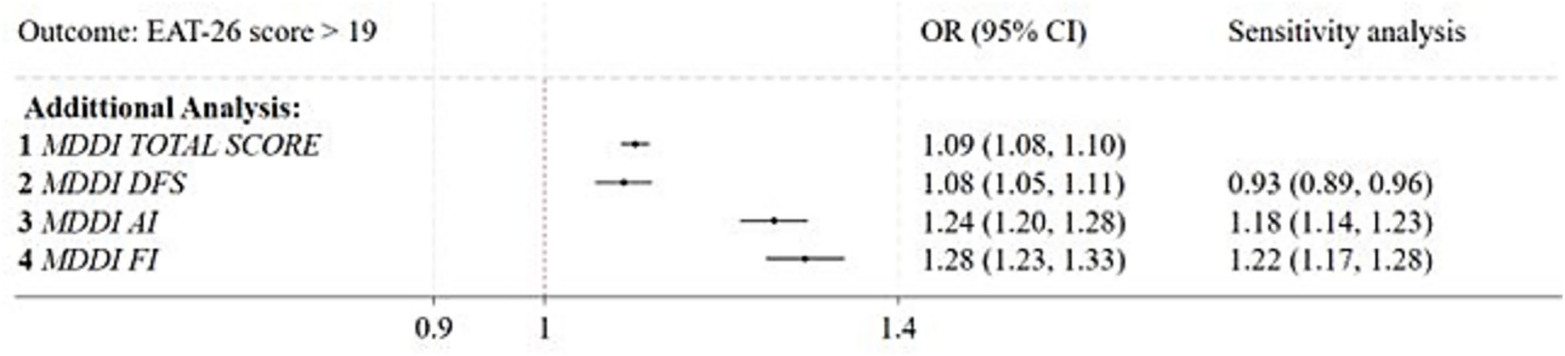
Forest plot of the multivariable logistic regression analysis evaluating factors associated with the risk of disordered eating attitudes. Diamonds represent the odds ratios (OR) and horizontal lines indicate the corresponding 95% confidence intervals (95% CI). The model was adjusted for age group, gender, educational level, degree course, smoking status, residence status, living situation, number of family members, and sport practice. MDDI: Muscle Dysmorphia Disorder Inventory (cut-off >39); Ref: Reference category.

In the main multivariable logistic regression model (Figure 1), high MD symptomatology (MDDI > 39) was associated with disordered eating risk (OR = 2.94; *p* < 0.001; 95% CI: 2.03–4.25). Furthermore, the model confirmed the influence of sociodemographic and lifestyle covariates previously observed. Significant inverse associations persisted for the 24–29 age group and for students living within social support systems (cohabiting with a partner, parents, or flatmates). Conversely, intense sports practice, higher educational attainment, and undisclosed gender remained positively associated with the outcome.

**Figure 2:**
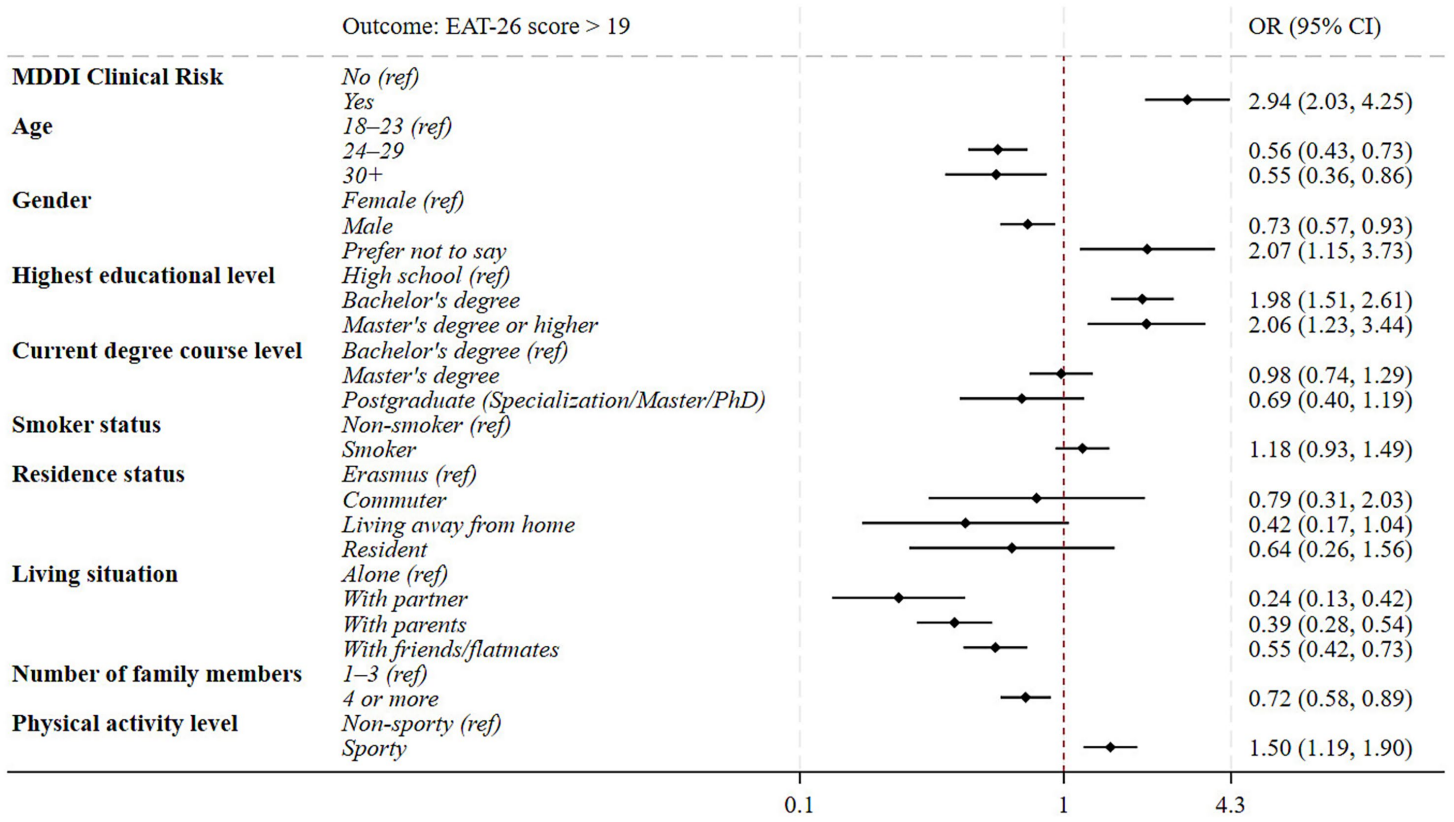
Forest plot illustrating the association between MDDI total score and subscales and disordered eating risk, individual models and sensitivity analysis including the three subscales. The plot displays the odds ratios (OR) derived from two analytical approaches: separate multivariable models for each subscale (single models) and a sensitivity analysis including all three subscales simultaneously. Diamonds represent the OR and horizontal lines indicate the 95% confidence intervals (95% CI). All models were adjusted for age group, gender, educational level, degree course, smoking status, residence status, living situation, number of family members, and sport practice. MDDI: Muscle Dysmorphia Disorder Inventory (cut-off >39); DFS: Drive for size; AI: Appearance intolerance; FI: Functional impairment.

Additional analyses provided granular insights into the specific drivers of the association (Figure 2). When modeled individually, the continuous MDDI total score and its three distinct subscales each exhibited a significant positive association with the outcome. However, the sensitivity analysis, considering all three subscales simultaneously, revealed divergent independent pathways. AI remained a significant positive correlation (OR = 1.18; *p* < 0.001; 95% CI: 1.14–1.23), as did FI (OR = 1.22; *p* < 0.001; 95% CI: 1.17–1.28). Conversely, the association with DFS was not confirmed by the sensitivity analysis, which instead revealed a significant inverse association (OR = 0.93; *p* < 0.05).

## 4 Discussion

The present study examined the complex relationship between ED symptomatology and the risk of EDs in a large university sample, providing new evidence on how specific psychopathological dimensions interact with socio-demographic variables and lifestyles. This interaction between lifestyle factors and health outcomes is consistent with broader biomedical research, which links systemic conditions and dysbiosis metabolites to accelerated aging (29). By dissecting these associations, we aim to provide a more granular understanding of the mechanisms linking body image concerns to disordered eating behaviors in the university population. Furthermore, the MDDI is one of the most robust and widely used instruments in the literature. Supporting this, a large systematic review and meta-analysis comparing bodybuilders with non-bodybuilder weight-trained individuals included only studies utilizing the muscle dysmorphia inventory (MDI) in the meta-analysis, demonstrating how standardized measures of MD perform in meta-analysis. Using the MDI subscales, bodybuilding was found to be associated with medium to large standardized mean differences in MD symptoms, with competitive bodybuilders scoring the highest.

Regarding psychological correlates, the severity of MD symptoms (measured with various instruments) is positively associated with anxiety, physical appearance-related social anxiety, depression, neuroticism, and perfectionism, and negatively associated with self-concept and self-esteem (30). The link between MD and perfectionism could be particularly relevant, given the generational trend of increasing multidimensional perfectionism in young adults, which potentially amplifies vulnerability to MD-like problems (31). In addition, a meta-analysis of MD-Eating Disorder (ED) relationships (using MDDI and other measures) reported a moderate aggregate positive correlation between MD symptoms and ED symptoms across 36 independent estimates (32).

### 4.1 Muscle dysmorphia and eating disorder risk: beyond categorical diagnosis

Our primary result confirms that the presence of ED symptoms (MDDI > 39) represents one of the strongest independent predictors for ED risk, with an adjusted OR of 2.94 in the multivariable model. This finding corroborates international literature that frames ED not merely as a body image disorder, but as a condition with profound behavioral overlaps with EDs, sharing traits of dietary rigidity and compulsive control (32, 33). The magnitude of this association suggests that the psychopathology of ED may act as a specific “gateway” to broader disordered eating. This may be influenced by the fact that sociocultural body ideals have converged toward a “lean-muscular” paradigm, characterized by a simultaneous pursuit of increased fat-free mass and reduced adiposity across both sexes. This evolution reflects a shift from traditional thinness or bulk toward an athletic esthetic that emphasizes hyper-muscularity in men and a toned, muscular-thin ideal in women (34, 35). These ideals, in particular, are largely conveyed by the media, including television, social platforms, and periodicals (36). Thus, individuals presenting rigid fitness goals should be routinely screened for eating disorders (37).

### 4.2 The differential impact of MDDI subscales: drive vs. distress

While univariate analyses suggested all MDDI subscales were predictive of ED risk, the simultaneous multivariable sensitivity analysis revealed a more nuanced picture. Only AI (OR of 1.18 per point) and FI (OR of 1.22 per point) were identified as true, independent drivers of this risk, with DFS showing a significant protective or inverse association (OR 0.93). These results suggest that the pursuit of muscular hypertrophy, when considered independently of body image dissatisfaction and impairment in social, occupational, and relational life, represents a protective factor against the risk of ED, likely because it is compatible with the desire to increase musculature for athletic or esthetic reasons, thereby excluding pathological implications (17, 38). Indeed, according to prior literature, drive for muscularity represents a normal, autonomously motivated pursuit that remains non-pathological provided it is devoid of clinically significant distress or functional impairment. The transition to MD is therefore defined by the emergence of maladaptive psychological preoccupation rather than the intensity of the muscularity drive itself (39, 40). Consequently, the pathological risk lies almost entirely in the inability to tolerate one’s body image, and the invalidating impact training has on daily life, rather than in the ambition for muscle growth itself (41).

### 4.3 Emerging adulthood and socio-environmental determinants

Analyzing demographic variables, a clear age-related gradient emerges. Students in the 18–23 age group present a significantly higher risk compared to their older peers (24–29 years: OR 0.56). This underscores the criticality of the emerging adulthood phase, characterized by university entry, detachment from the family, and identity renegotiation (42, 43). Younger students appear less equipped to handle academic and social pressure, potentially resorting to body control as a dysfunctional coping mechanism to regain a sense of agency (44). Furthermore, our data regarding living arrangements provides compelling evidence for role of the social environment. Living alone confirms itself as a primary risk factor, while cohabiting with a partner (OR 0.24) or parents (OR 0.39) offers robust protection. Additionally, belonging to families with 4 or more members reduces risk by 28% (OR 0.72). This aligns with evidence suggesting that greater emotional attachment and dependence on parents are associated with an increased risk of MD, potentially due to parental criticism and social pressure linked to expectations. Such findings primarily involve samples of individuals during the critical adolescent period. However, in male bodybuilders (many in the young-adult range), being at risk for MD was specifically linked to an insecure-avoidant adult attachment style (45, 46), which implies suboptimal parental bonding during childhood and is characterized by a strong desire for independence, emotional distance, and discomfort with intimacy. In contrast, a secure attachment style, associated with a more positive self-image and greater trust in one’s relationships, appeared negatively associated to MD (47). In addition, our results could be read in light of “protective commensality” hypothesis, according to which the physical presence of others during meals and a direct social support network seem to act as a “buffer” against MD and EDs, which tend to proliferate in the isolation and lack of social supervision typical of many off-campus students (48–50).

### 4.4 Gender nuances and the burden of minority stress

While the binary male–female comparison confirmed the known female prevalence in EAT-26 risk (with males showing a protective OR of 0.73 in the adjusted model) (51), participants preferring not to disclose their gender present a statistically significant risk more than double (OR 2.07) that of females. These results require cautious interpretation, as the category of participants who chose not to disclose their gender may also reflect privacy concerns. However, given the complete anonymity of the survey, which mitigated privacy-related non-disclosure bias, and although we can assume that a significant portion of respondents who chose not to specify their gender are participants who did not identify within the gender binary, these subjects were categorized as gender-diverse. Indeed, the act of concealing one’s gender identity is a significant stressor, aligning with minority stress theory. Research suggests that these behaviors often serve as maladaptive coping strategies for identity-related distress or as ways to alter body characteristics when social affirmation is lacking (52, 53). Furthermore, it was highlighted that paranoid ideation—a thought pattern characterized by suspicion and distrust toward others, highly prevalent among individuals who conceal their gender and, generally, correlated with all forms of minority stress—is associated with muscle dysmorphia or bigorexia (54, 55). Therefore, individuals who choose not to disclose their identity are considered high-risk, as they typically experience heightened internalized stigma and appearance-related anxiety.

### 4.5 Lifestyles: the paradox of sport and smoking

Finally, the analysis of lifestyles reveals two contrasting dynamics. On one hand, smoking status, despite showing a crude positive association in univariate analyses, loses significance in the multivariable model (OR 1.18, CI includes 1). This suggests that smoking is not a direct driver of EDs, but rather a behavior that co-occurs with other stress factors or anxious personality traits that are the true culprits of risk (56). On the other hand, intense sports practice remains significantly associated with a higher risk of EDs (OR 1.50) even after adjustment. This highlights the paradox of physical activity in this population: while generally healthy, in a subgroup of students, frequent exercise is not motivated by well-being but becomes compensatory or compulsive practice (57). This aligns with the construct of “Orthorexia Nervosa” and “Athletic Anorexia,” where the gym becomes a venue for calorie purging rather than fitness building (58).

### 4.6 Limitations

While this study provides significant insights into the interplay between ED and ED risk among university students, several limitations must be acknowledged when interpreting the results.

First and foremost, the cross-sectional design 1 precludes the determination of causality. While we observed a strong association where ED symptoms predict disordered eating risk, we cannot rule out bidirectional effects or the possibility that both conditions will emerge simultaneously from shared underlying transdiagnostic vulnerabilities, such as perfectionism or emotional dysregulation.

Second, the reliance on self-reported questionnaires (EAT-26 and MDDI) introduces the potential for social desirability bias. This is particularly relevant in the context of “fitness culture,” where excessive exercise and rigid dietary control are often socially valorized rather than recognized as pathological, potentially leading to underreporting of distress.

Third, although the sample size was robust (*N* = 2001), the participants were recruited from a single university context. This may limit the generalizability of findings to non-student young adults or those from different cultural or socio-economic backgrounds who may not face the specific academic and social pressures of university life.

Fourth, regarding the gender analysis, although the finding regarding participants who “preferred not to say” their gender was statistically significant (OR = 2.07), the sample size of this subgroup was relatively small (*n* = 58). Consequently, the wide confidence intervals suggest that while the elevated risk is clear, the precise magnitude of this effect requires replication in larger, specifically targeted cohorts.

Fifth, continuous data for age and anthropometric measurements were not collected. Age was recorded categorically to maximize perceived anonymity. Furthermore, weight and height were deliberately omitted. Self-reported anthropometric data in populations at risk of eating disorders are notoriously subject to severe reporting and social desirability biases. Consequently, Body Mass Index (BMI) could not be calculated, leaving it as a recognized unmeasured confounder in our analyses (59).

Sixth, a significant limitation of this research is the lack of a clinical comparison group. As the study was conducted on a general university population, the findings reflect screening risks rather than confirmed clinical diagnoses. The absence of a clinical sample prevents us from comparing the symptom profiles of sub-clinical students with those of patients already diagnosed with MD or EDs, which would have provided deeper insight into the severity and progression of these pathologies. Additionally, our analysis did not include the assessment of common psychiatric comorbidities such as anxiety, depression, or compulsive behaviors. Given that these conditions frequently interact with both muscle dysmorphia and eating disorders, their absence as covariates represents a limitation in the interpretation of the observed associations. Future studies should aim to integrate these psychological dimensions to provide a more comprehensive adjustment for potential confounders.

Finally, it should be noted that while the EAT-26 is a validated screening tool, it does not provide a formal clinical diagnosis aligned with all DSM-5 criteria. Given the substantial size of our cohort, the use of a more comprehensive diagnostic instrument allowing for the analysis of specific symptom profiles or subscales would have been highly valuable. Future research should consider incorporating tools that allow for a more nuanced investigation of diagnostic dimensions to better characterize the transition between MD and specific ED subtypes.

## 5 Conclusion

Research substantiates that MD, delineated by the dimensions of AI and FI, serves as an independent prognosticator of EDs risk among university students. The delineation of a high-risk phenotype (characterized by younger age, social isolation, and intensive athletic participation) necessitates appropriate screening procedures. Pathological manifestation is contingent upon the confluence of the pursuit of muscularity and concurrent psychological distress, thereby underscoring the critical importance of social support. Practical implications advocate for a paradigm shift in university wellness programs, moving from conventional, nutrition-centric screenings toward refined psychological assessments that prioritize MD symptomatology. An MDDI score exceeding 39 is posited as a potent independent predictor of ED risk; accordingly, healthcare professionals should focus attention on the AI and FI subscales, as these constitute the principal drivers of pathological eating behaviors, more so than the isolated pursuit of muscularity. Prevention strategies mandate tailoring for individuals within the 18–23 age cohort and those experiencing social isolation. Prospective research should incorporate longitudinal investigations to elucidate the causal nexus between MD and EDs, mitigating the limitations inherent in cross-sectional methodologies, and integrate clinical comparison groups alongside diagnostic instruments calibrated with DSM-5 criteria. Furthermore, the expansion of the analytical framework to encompass psychiatric comorbidities (e.g., perfectionism, anxiety, depression) and objective anthropometric indices is essential for a comprehensive understanding of transdiagnostic mechanisms and socio-environmental determinants.

## Data Availability

Data presented in this study may be available upon reasonable request and approval by the research team.
